# Game Theory of Mind

**DOI:** 10.1371/journal.pcbi.1000254

**Published:** 2008-12-26

**Authors:** Wako Yoshida, Ray J. Dolan, Karl J. Friston

**Affiliations:** The Wellcome Trust Centre for Neuroimaging, University College London, United Kingdom; John Radcliffe Hospital, United Kingdom

## Abstract

This paper introduces a model of ‘theory of mind’, namely, how we represent the intentions and goals of others to optimise our mutual interactions. We draw on ideas from optimum control and game theory to provide a ‘game theory of mind’. First, we consider the representations of goals in terms of value functions that are prescribed by utility or rewards. Critically, the joint value functions and ensuing behaviour are optimised recursively, under the assumption that I represent your value function, your representation of mine, your representation of my representation of yours, and so on ad infinitum. However, if we assume that the degree of recursion is bounded, then players need to estimate the opponent's degree of recursion (i.e., sophistication) to respond optimally. This induces a problem of inferring the opponent's sophistication, given behavioural exchanges. We show it is possible to deduce whether players make inferences about each other and quantify their sophistication on the basis of choices in sequential games. This rests on comparing generative models of choices with, and without, inference. Model comparison is demonstrated using simulated and real data from a ‘stag-hunt’. Finally, we note that exactly the same sophisticated behaviour can be achieved by optimising the utility function itself (through prosocial utility), producing unsophisticated but apparently altruistic agents. This may be relevant ethologically in hierarchal game theory and coevolution.

## Author Summary

The ability to work out what other people are thinking is essential for effective social interactions, be they cooperative or competitive. A widely used example is cooperative hunting: large prey is difficult to catch alone, but we can circumvent this by cooperating with others. However, hunting can pit *private* goals to catch smaller prey that can be caught alone against *mutually beneficial* goals that require *cooperation*. Understanding how we work out optimal strategies that balance cooperation and competition has remained a central puzzle in game theory. Exploiting insights from computer science and behavioural economics, we suggest a model of ‘theory of mind’ using ‘recursive sophistication’ in which my model of your goals includes a model of your model of my goals, and so on ad infinitum. By studying experimental data in which people played a computer-based group hunting game, we show that the model offers a good account of individual decisions in this context, suggesting that such a formal ‘theory of mind’ model can cast light on how people build internal representations of other people in social interactions.

## Introduction

This paper is concerned with modelling the intentions and goals of others in the context of social interactions; in other words, how do we represent the behaviour of others in order to optimise our own behaviour? Its aim is to elaborate a simple model of ‘theory of mind’ [1],[2] that can be inverted to make inferences about the likely strategies subjects adopt in cooperative games. Critically, as these strategies entail inference about other players, this means the model itself has to embed inference about others. The model tries to reduce the problem of representing the goals of others to its bare essentials by drawing from optimum control theory and game theory.

We consider ‘theory of mind’ at two levels. The first concerns how the goals and intentions of another agent or player are *represented*. We use optimum control theory to reduce the problem to representing value-functions of the states that players can be in. These value-functions prescribe optimal behaviours and are specified by the utility, payoff or reward associated with navigating these states. However, the value-function of one player depends on the behaviour of another and, implicitly, their value-function. This induces a second level of theory of mind; namely the problem of *inference* on another's value-function. The particular problem that arises here is that inferring on another player who is inferring your value-function leads to an infinite regress. We resolve this dilemma by invoking the idea of ‘bounded rationality’ [3],[4] to constrain inference through priors. This subverts the pitfall of infinite regress and enables tractable inference about the ‘type’ of player one is playing with.

Our paper comprises three sections. The first deals with a theoretical formulation of ‘theory of mind’. This section describes the basics of representing goals in terms of high-order value-functions and policies; it then considers inferring the unknown order of an opponent's value-function (i.e., sophistication or type) and introduces priors on their sophistication that finesse this inference. In the second section, we apply the model to empirical behavioural data, obtained while subjects played a sequential game, namely a ‘stag-hunt’. We compare different models of behaviour to quantify the likelihood that players are making inferences about each other and their degree of sophistication. In the final section, we revisit optimisation of behaviour under inferential theory of mind and note that one can get exactly the same equilibrium behaviour without inference, if the utility or payoff functions are themselves optimised. The ensuing utility functions have interesting properties that speak to a principled emergence of ‘inequality aversion’ [5] and ‘types’ in social game theory. We discuss the implications of this in the context of evolution and hierarchical game theory.

## Model

Here, we describe the optimal value-function from control theory, its evaluation in the context of one agent and then generalise the model for interacting agents. This furnishes models that can be compared using observed actions in sequential games. These models differ in the degree of recursion used to construct one agent's value-function, as a function of another's. This degree or order is bounded by the *sophistication* of agents, which determines their optimum *strategy*; i.e., the optimum policy given the policy of the opponent. Note that we will refer to the policy on the space of policies as a strategy and reserve policy for transitions on the space of states. Effectively, we are dealing with a policy hierarchy where we call a second-level policy a strategy. We then address inference on the policy another agent is using and optimisation under the implicit unobservable states. We explore these schemes using a stag-hunt, a game with two Nash equilibria, one that is risk-dominant and another that is payoff-dominant. This is important because we show that the transition from one to the other rests on sophisticated, high-order representations of an opponent's value-function.

### Policies and Value Functions

Let the admissible states of an agent be the set 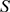, where the state at any time or trial 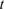 is 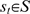. We consider environments under Markov assumptions, where 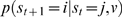 is the probability of going from state *j* to state *i*. This transition probability defines the agent's policy as a function of value 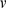. We can summarise this policy in terms of a matrix 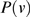, with elements 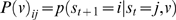. In what follows, will use 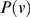 to denote a probability transition matrix that depends on 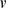 and 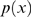 for a probability on 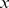. The value of a state is defined as utility or payoff, 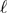 expected under iterations of the policy and can be defined recursively as
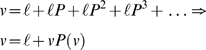
(1)The notion of value assumes the existence of a state-dependent quantity that the agent optimises by moving from one state to another. In Markov environments with *n* = |*S*| states, the value over states, encoded in the row vector *v*∈ℜ^1×*n*^, is simply the payoff at the current state ℓ∈ℜ^1×*n*^ plus the payoff expected on the next move, ℓ*P*, the subsequent move ℓ*P* ^2^ and so on. In short, value is the reward expected in the future and satisfies the Bellman equation [6] from optimal control theory; this is the standard equation of dynamic programming
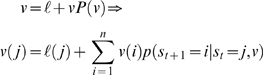
(2)We will assume a policy is fully specified by value and takes the form
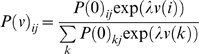
(3a)Under this assumption, value plays the role of an energy function, where *λ* is an inverse temperature or precision; assumed to take a value of one in the simulations below. Using the formalism of Todorov [7], the matrix *P*(0) encodes autonomous (uncontrolled) transitions that would occur when, 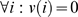. These probabilities define admissible transitions and the nature of the state-space the agent operates in, where inadmissible transitions are encoded with *P*(0)*_ij_* = 0. The uncontrolled transition probability matrix *P*(0) plays an important role in the general setting of Markov decision processes (MDP). This is because certain transitions may not be allowed (e.g., going though a wall). Furthermore, there may be transitions, even in the absence of control, which the agent is obliged to make (e.g., getting older). These constraints and obligatory transitions are encoded in *P*(0). The reader is encouraged to read Ref. [7] for a useful treatment of optimal control problems and related approximation strategies.

Equation 3a is intuitive, in that admissible states with relatively high value will be visited with greater probability. Under some fairly sensible assumptions about the utility function (i.e., assuming a control cost based on the divergence between controlled and uncontrolled transition probabilities), Equation 3 is the optimum policy.

This policy connects our generative model of action to economics and behavioural game theory [8], where the softmax or logit function (Equation 3) is a ubiquitous model of transitions under value or attraction; for example, a logit response rule is used to map attractions, 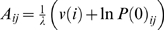 to transition probabilities:
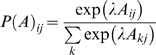
(3b)In this context, *λ* is known as response *sensitivity*; see Camerer [8] for details. Furthermore, a logit mapping is also consistent with stochastic perturbations of value, which leads to quantal response equilibria (QRE). QRE are a game-theoretical formulation [9], which converges to the Nash equilibrium when *λ* goes to infinity. In most applications, it is assumed that perturbations are drawn from an extreme value distribution, yielding the familiar and convenient logit choice probabilities in Equation 3 (see [10] for details). Here, *λ* relates to precision of random fluctuations on value.

Critically, Equation 3 prescribes a probabilistic policy that is necessary to define the likelihood of observed behaviour for model comparison. Under this fixed-form policy, the problem reduces to optimising the value-function (i.e., solving the nonlinear self-consistent Bellman equations). These are solved simply and quickly by using a Robbins-Monro or stochastic iteration algorithm [11]

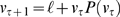
(4)At convergence, 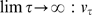 becomes the optimal value-function, which is an analytic function of payoff; 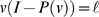. From now on, we will assume 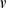 is the solution to the relevant Bellman equation. This provides an optimum value-function for any state-space and associated payoff, encoded in a ‘game’.

Clearly, this is not the only way to model behaviour. However, the Todorov formalism greatly simplifies the learning problem and provides closed-form solutions for optimum value: In treatments based on Markov decision processes, in which the state transition matrix depends on an action, *both* the value-function and policy are optimised iteratively. However, by assuming that value effectively prescribes the transition probabilities (Equation 3), we do not have to define ‘action’ and avoid having to optimise the policy *per se*. Furthermore, as the optimal value is well-defined we do not have to worry about learning the value-function. In other words, because the value-function can be derived analytically from the loss-function (irrespective of the value-learning scheme employed by the agent), we do not need to model *how* the agent comes to acquire it; provided it learns the veridical value-function (which in many games is reasonably straightforward). This learning could use dynamic programming [12], or Q-learning [13], or any biologically plausible scheme.

### A Toy Example

**Figure 1:**
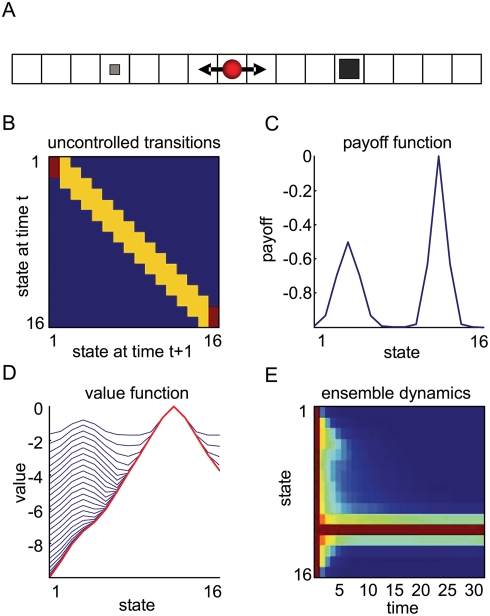
Toy example using a one-dimensional maze. (A) The agent (red circle) moves to the adjacent states from any given state to reach a goal. There are two goals, where the agent obtains a small payoff (small square at state 4) or a big payoff (big square at state 12). (B) The uncontrolled state transition matrix. (C) The payoff-function over the states with a local and global maximum. (D) Iterative approximations to the optimal value-function. In early iterations, the value-function is relatively flat and shows a high value at the local maximum. With a sufficient number of iterations, *τ*≥24, the value-function converges to the optimum (the red line) which induces paths toward the global maximum at state 12. (E) The dynamics of an ensemble density, under the optimal value-function. The density is uniform on state-space at the beginning, *t* = 1, and develops a sharp peak at the global maximum over time.

The example in Figure 1 illustrates the nature and role of the quantities described above. We used a one-dimensional state-space with *n* = 16 states, where an agent can move only to adjacent states (Figure 1A). This restriction is encoded in the uncontrolled transition probabilities. We assumed the agent is equally likely to move, or not move, when uncontrolled; i.e., the probability of remaining in a state is equal to the sum of transitions to other states (Figure 1B). To make things interesting, we considered a payoff function that has two maxima; a local maximum at state four and the global maximum at state twelve (Figure 1C). In effect, this means the optimum policy has to escape the local maximum to reach the global maximum. Figure 1D shows the successive value-function approximations as Equation 4 is iterated from *τ* = 1 to 32. Initially, the local maximum captures state-trajectories but as the value-function converges to the optimal value-function, it draws paths through the local maximum, toward the global maximum. Instead of showing example trajectories under the optimal value-function, we shows the density of an ensemble of agents, *ρ*(*s*,*t*), as a function of time, starting with a uniform distribution on state-space, *ρ*(*s*,0) = 1/*n* (Figure 1E). The ensemble density dynamics are given simply by 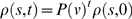. It can be seen that nearly all agents have found their goal by about *t* = 18 ‘moves’.

In summary, we can compute an optimal value-function for any game, *G*(ℓ,*P*(0)) specified in terms of payoffs and constraints. This function specifies the conditional transition probabilities that define an agent's policy, in terms of the probability of emitting a sequence of moves or state-transitions. In the next section, we examine how value-functions are elaborated when several agents play the same game.

### Games and Multiple Agents

When dealing with two agents the state-space becomes the Cartesian product of the admissible states of both agents, *S* = *S*_1_×*S*_2_ (Note that all that follows can be extended easily to over *m* agents.). This means that the payoff 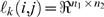 and value 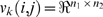 are defined on a joint-space for each agent *k*. The payoff for the first agent ℓ_1_(*i*, *j*) occurs when it is in state *i* and the second is in state *j*. This can induce cooperation or competition, unless the payoff for one agent does not depend on the state of the other: i.e., ∀*j*,*k* : ℓ_1_(*i*, *j*) = ℓ_1_(*i*, *k*). Furthermore, the uncontrolled probabilities for one agent now become a function of the other agent's value, because one agent cannot control the other. This presents an interesting issue of how one agent represents the policy of the other.

In what follows, we consider policies that are specified by an order: first-order policies discount the policies of other agents (i.e., I will ignore your goals). Second-order policies are optimised under the assumption that you are using a first-order policy (i.e., you are ignoring my goals). Third-order policies pertain when I assume that you assume I am using a first-order policy and so on. This construction is interesting, because it leads to an infinite regress: I model your value-function but your value-function models mine, which includes my model of yours, which includes my model of your model of mine and so on *ad infinitum*. We will denote the *i*-th order value-function for the *k*-th agent by 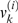. We now consider how to compute these value-functions.

### Sequential Games

In a sequential game, each agent takes a turn in a fixed order. Let player one move first. Here, the transition probabilities 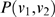 now cover the Cartesian product 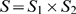 of the states of both agents and the joint transition-matrix 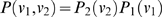 factorises into agent-specific terms. These are given by
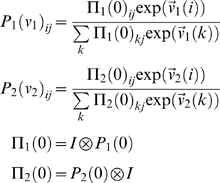
(5)where Π*_k_*(0) specifies uncontrolled transitions in the joint-space, given the uncontrolled transitions *P_k_*(0) in the space of the *k*-th agent. Their construction using the Kronecker tensor product ⊗ ensures that the transition of one agent does not change the state of the other. Furthermore, it assumes that the uncontrolled transitions of one agent do not depend on the state of the other; they depend only on the uncontrolled transitions *P_k_*(0) among the *k*-th agent's states. The row vectors 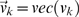 are the vectorised versions of the two dimensional value-functions for the *k*-th agent, covering the joint states. We will use a similar notation for the payoffs, 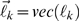. Critically, both agents have a value-function on every joint-state but can only change their own state. These value-functions can now be evaluated through recursive solutions of the Bellman equations
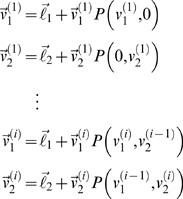
(6)This provides a simple way to evaluate the optimal value-functions for both agents, to any arbitrary order. The optimal value-function for the first agent, when the second is using 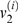 is 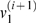. Similarly, the optimal value under 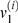 for the second is 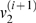. It can be seen that under an optimum strategy (i.e., a second-level policy) each agent should increase its order over the other until a QRE obtains when 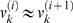 for both agents. However, it is interesting to consider equilibria under non-optimal strategies, when both agents use low-order policies in the mistaken belief that the other agent is using an even lower order. It is easy to construct examples where low-order strategies result in risk-dominant policies, which turn into payoff-dominant policies as high-order strategies are employed; as illustrated next.

### A Stag-Hunt

**Figure 2:**
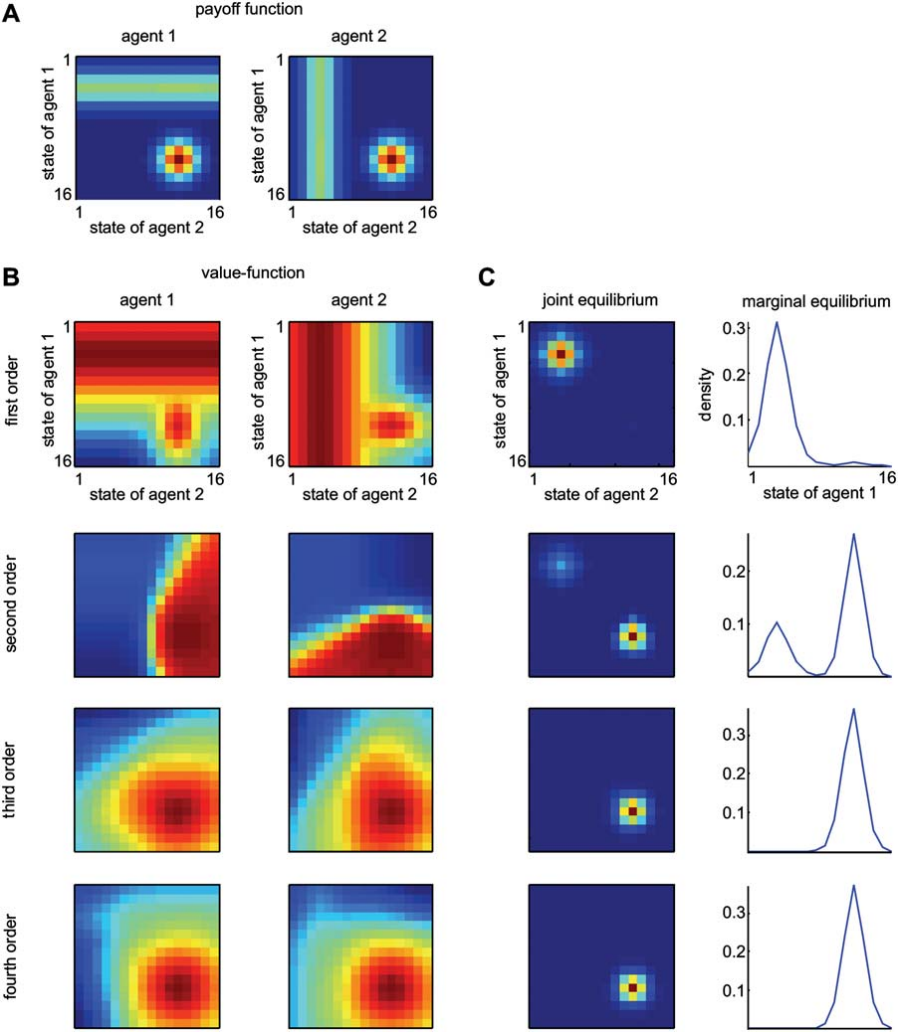
Stag-hunt game with two agents. (A) The payoff-functions for the first (the left panel) and the second agent (the right panel) over the joint state-space. The red colour indicates a higher payoff. The payoff of the ‘stag’ (state 12) is higher than the ‘rabbit’ (state 4). (B) Optimal value-functions of first, second, third and fourth order (from the top to the bottom) for both agents. The low-order value-functions focus on the risk-dominant states, while high-order functions lead to payoff dominant states that require cooperation. (C) The equilibrium densities of an ensemble of agents after 128 moves, when both agents use matched value-functions in (B). The left and right panels show the joint and marginal equilibrium densities over the joint state-space and the state of the first agent, respectively.

In this example, we used a simple two-player stag-hunt game where two hunters can either jointly hunt a stag or pursue a rabbit independently [14]. Table 1 provides the respective payoffs for this game as a normal form representation. If an agent hunts a stag, he must have the cooperation of his partner in order to succeed. An agent can catch a rabbit by himself, but a rabbit is worth less than a stag. This furnishes two pure-strategy equilibria: one is risk-dominant with low-payoff states that can be attained without cooperation (i.e., catching a rabbit) and the other is payoff dominant; high-payoff states that require cooperation (i.e., catching a stag). We assumed the state-space of each agent is one-dimensional with *n*_1_ = *n*_2_ = 16 possible states. This allows us to depict the value-functions on the joint space as two-dimensional images. The dimensionality of the state-space is not really important; however, a low-dimensional space imposes sparsity on the transition matrices, because only a small number of neighbouring states can be visited from any given state. These constraints reduce the computational load considerably. The ‘rabbit’ and ‘stag’ do not move; the rabbit is at state four and the stag at state twelve. The key difference is that the payoff for the ‘stag’ is accessed only when both players occupy that state (or nearby), whereas the payoff for the ‘rabbit’ does not depend on the other agent's state. Figure 2A shows the characteristic payoff functions for both agents. The ensuing value-functions for the order *i* = 1,…,4 from Equation 6 are shown in Figure 2B. It can be seen that first-order strategies defined by 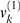 regard the ‘stag’ as valuable, but only when the other agent is positioned appropriately. Conversely, high-order strategies focus exclusively on the stag. As one might intuit, the equilibrium densities of an ensemble of agents acting under first or high-order strategies have qualitatively different forms. Low-order strategies result in both agents hunting the ‘rabbit’ and high-order schemes lead to a cooperative focus on the ‘stag’. Figure 2C shows the joint and marginal equilibrium ensemble densities 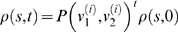 for *t* = 128 (i.e., after 128 moves) and a uniform starting distribution; for matched strategies, *i* = 1,…,4.

**Table 1:** Normal-form representation of a stag-hunt in terms of payoffs in which the following relations hold: *A*>*B*≥*C*>*D* and *a*>*b*≥*c*>*d*.

		Hunter 2	
		Stag	Rabbit
**Hunter 1**	**Stag**	A, a	C, b
	**Rabbit**	B, c	D, d

Upper-case letters represent the payoffs for the first hunter and lower-case letters represent the payoffs for the second.

### Inferring an Agent's Strategy

**Figure 3:**
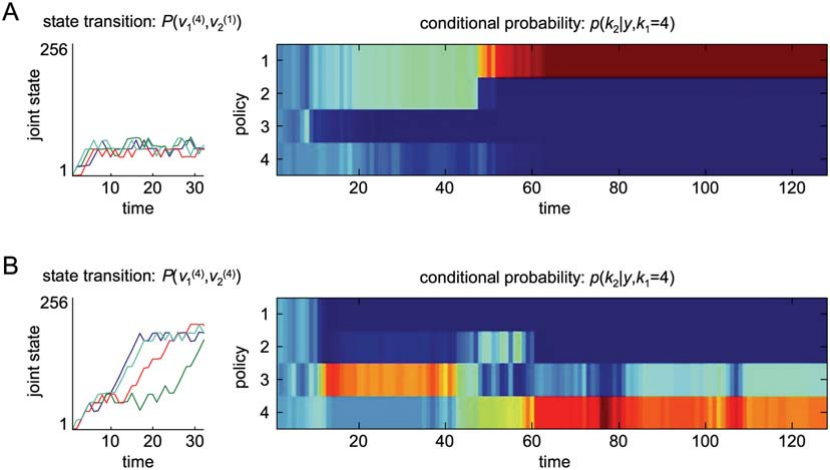
Inference on agent's strategy in the stag-hunt game. We assumed agents used unmatched strategies, in which the first agent used a fourth order strategy and the second agent used a first order strategy (A), and matched strategies - both agents used the fourth order strategy (B). The left panels show four state trajectories of 32 moves simulated using (or generated from) value-functions in Figure 2B. The right panels show the conditional probabilities of the second agent's strategy over a model-space of 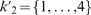 as a function of time.

In contrast to single-player games, polices in multi-player games have an order, where selecting the optimal order depends on the opponent. This means we have to consider how players evaluate the probability that an opponent is using a particular policy or how we, as experimenters, make inferences about the policies players use during sequential games. This can be done using the evidence for a particular policy, given the choices made. In the course of a game, the trajectory of choices or states *y* = *s*_1_,*s*_2_,…,*s_T_* is observed directly such that, under Markov assumptions
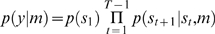
(7)Where *m*∈*M* represents a model of the agents and entails the quantities needed to specify their policies. The probability of a particular model, under flat priors on the models, is simply
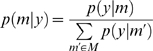
(8)To illustrate inference on strategy, consider the situation in which the strategy (i.e., the policy order *k*_1_) of the first agent is known. This could be me and I might be trying to infer your policy, to optimise mine; or the first agent could be a computer and the second a subject, whose policy we are trying to infer experimentally. In this context, the choices are the sequence of joint-states over trials, *y*∈*S*, where there are *n*_1_×*n*_2_ possible states; note that each joint state subsumes both ‘moves’ of each agent. From Equation 8 we can evaluate the probability of the second agent's strategy, under the assumption it entails a fixed and ‘pure’ policy of order *k*_2_

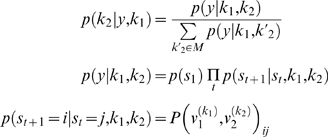
(9)Here, the model is specified by the unknown policy order, *m* = *k*_2_ of the second agent. Equation 9 uses the joint transition probabilities on the moves of all players; however, one gets exactly the same result using just the moves and transition matrix from the player in question. This is because, the contributions of the other players cancel, when the evidence is normalised. We use the redundant form in Equation 9 so that it can be related more easily to inference on the joint strategies of all agents in Equation 8. An example of this inference is provided in Figure 3. In Figure 3A and 3B, we used unmatched and matched strategies to generate samples using the probability transition matrices 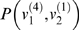 and 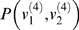; starting in the first state (i.e., both agents in state 1) respectively. These simulated games comprised four consecutive 32-move trials of the stag-hunt game specified in Figure 2. The ensuing state trajectories are shown in the left panels. We then inverted the sequence using Equation 9 and a model-space of 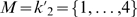. The results for *T* = 1,…,128 are shown in the right panels. For both simulations, the correct strategy discloses itself after about sixty moves, in terms of conditional inference on the second agent's policy. It takes this number of trials because, initially, the path in joint state-space is ambiguous; as it moves towards both the rabbit and stag.

### Bounded Rationality

We have seen how an *N*-player game is specified completely by a set of utility functions and a set of constraints on state-transitions. These two quantities define, recursively, optimal value-functions, 
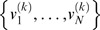
 of increasing order and their implicit policies. Given these policies, one can infer the strategies employed by agents, in terms of which policies they are using, given a sequence of transitions. In two-player games, when the opponent uses policy *k*, the optimum strategy is to use policy *k*+1. This formulation accounts for the representation of another's goals and optimising both policies and strategies. However, it induces a problem; to optimise ones own strategy, one has to know the opponent's policy. Under rationality assumptions, this is not really a problem because rational players will, by induction, use policies of sufficiently high order to ensure 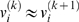. This is because each player will use a policy with an order that is greater than the opponent and knows a rational opponent will do the same. The interesting issues arise when we consider bounds or constraints on the strategies available to each player and their prior expectations about these constraints.

Here, we deal with optimisation under bounded rationality [4] that obliges players to make inferences about each other. We consider bounds, or constraints, that lead to inference on the opponent's strategy. As intimated above, it is these bounds that lead to interesting interactions between players and properly accommodate the fact that real players do not have unbounded computing resources to attain a QRE by using 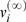. These constraints are formulated in terms of the policy *k_i_* of the *i*-th player, which specifies the corresponding value-function and policy 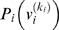. The constraints we consider are:

- The *i*-th player uses an approximate conditional density *q_i_*(*k_j_*) on the strategy of the *j*-th player that is a point mass at the conditional mode, 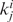.
- Each player has priors *p_i_*(*k_j_*), which place an upper bound on the opponents sophistication; ∀*k_j_*>*K_i_* : *p_i_*(*k_j_*) = 0

These assumptions have a number of important implications. First, because *q_i_*(*k_j_*) is a point mass at the mode 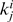, each player will assume every other player is using a pure strategy, as opposed to a strategy based on a mixture of value-functions. Second, under this assumption, each player will respond optimally with another *pure* strategy, 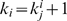. Third, because there is an upper bound on 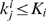 imposed by an agent's priors, they will never call upon strategies more sophisticated than *k_i_* = *K_i_*+1. In this way, *K_i_* bounds both the prior assumptions about other players and the sophistication of the player *per se*. This defines a ‘type’ of player [15] and is the central feature of the bounded rationality under which this model is developed. Critically, type is an attribute of a player's prior assumptions about others. The nature of this bound means that any player cannot represent the goals or intentions of another player who is more sophisticated; in other words, it precludes any player ‘knowing the mind of God’ [16].

### Representing the Goals of Another

**Figure 4:**
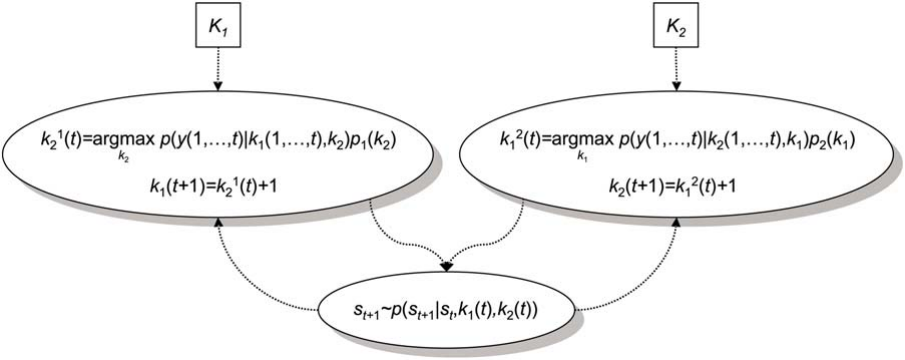
Schematic detailing inference on an opponent's strategy.

Under flat priors on the bounded support of the priors *p_i_*(*k_j_*), the mode can be updated with each move using Equation 9. Here, player one would approximate the conditional density on the opponent's strategy with the mode
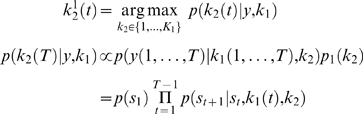
(10)And optimise its strategy accordingly, by using 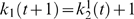. This scheme assumes the opponent uses a fixed strategy and consequently accumulates evidence for each strategy over the duration of the game. Figure 4 illustrates the conditional dependencies of the choices and strategies; it tries to highlight the role of the upper bounds in precluding recursive escalation of *k_i_*(*t*). Note, that although each player assumes the other is using a stationary strategy, the players own policy is updated after every move.

**Figure 5:**
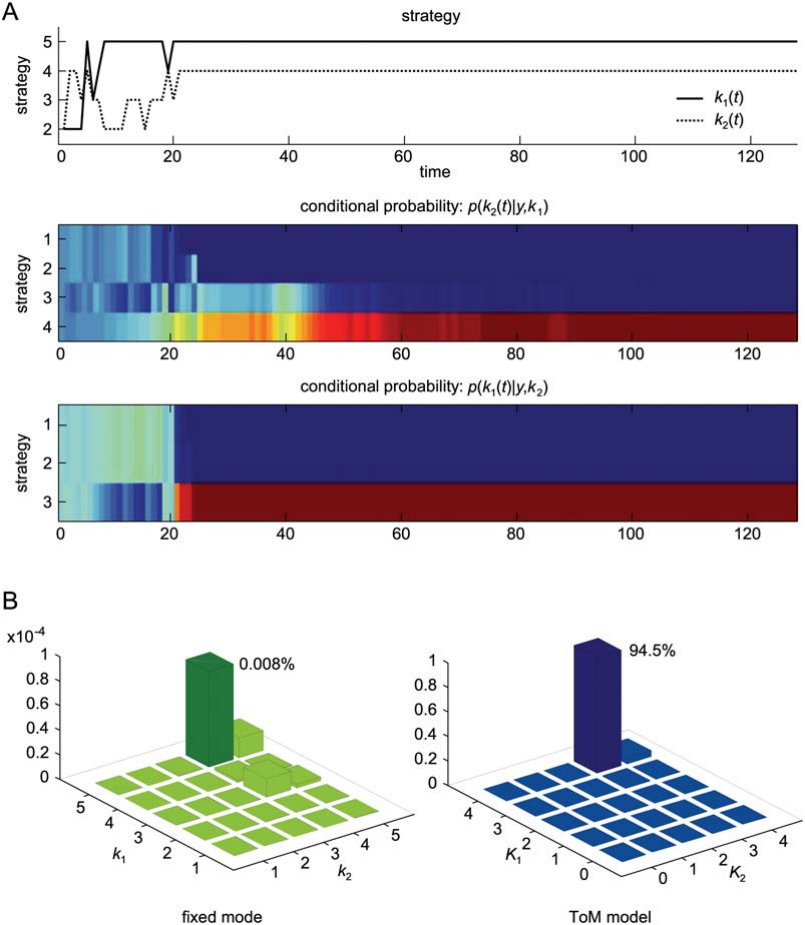
Inference on opponent's types in the stag-hunt game. Two players with asymmetric types *K*_1_ = 4 and *K*_2_ = 3 used an optimum strategy based on the inferred opponent's strategy. (A) The top panel shows the strategies of both agents over time. The middle and bottom panels show the implicit densities of the opponent's strategy for the first and the second player, respectively. The densities for both agents converge on the correct opponent's strategies after around 20 moves. (B) The posterior probabilities over fixed and theory of mind (ToM) models. The left graph shows the likelihood over fixed models using *k*_1_,*k*_2_ = 1,…,5 and the right graph shows the likelihood of ToM models with *K*_1_,*K*_2_ = 0,…,4. The veridical model (dark blue bar) shows model with the maximum likelihood, among 50 models.

Figure 5A shows a realization of a simulated stag-hunt using two types of player with asymmetric bounds *K*_1_ = 4 and *K*_2_ = 3 (both starting with *k_i_*(1) = 1). Both players strive for an optimum strategy using Equation 10. We generated four consecutive 32-move trials; 128 trials in total, starting in the first state with both agents in state one. After 20 moves, the first, more sophisticated, player has properly inferred the upper bound of the second and plays at one level above it. The second player has also optimised its strategy, which is sufficiently sophisticated to support cooperative play. The lower panels show the implicit density on the opponent's strategy, *p*(*k*_2_|*y*,*k*_1_); similarly for the second player. The mode of this density is 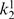 in Equation 10.

### Inferring Theory of Mind

We conclude this section by asking if we, as experimenters, can infer *post hoc* on the ‘type’ of players, given just their choice behaviours. This is relatively simple and entails accumulating evidence for different models in exactly the same way that the players do. We will consider fixed-strategy models in which both players use a fixed *k_i_* or theory of mind models, in which players infer on each other, to optimise *k_i_*(*t*) after each move. The motivation for considering fixed models is that they provide a reference model, under which the policy is not updated and therefore there is no need to infer the opponent's policy. Fixed models also relate to an alternative [prosocial] scheme for optimising behaviour, reviewed in the discussion. The evidence for fixed models is
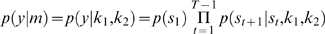
(11)Whereas the evidence for theory of mind models is
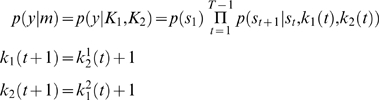
(12)where 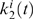 are inferred under the appropriate priors specified by *K_i_*. The key difference between these models is that the policy changes adaptively in the theory of mind model, in contrast to the fixed model.

Under flat model priors, the posterior, *p*(*m_i_*|*y*) (Equation 8) can be used for inference on model-space. We computed the posterior probabilities of fifty models, using Equation 11 and 12. Half of these models were fixed models using *k*_1_,*k*_2_ = 1,…,5 and the remaining were theory of mind models with *K*_1_,*K*_2_ = 0,…,4. Figure 5B shows the results of this model comparison using the simulated data shown in Figure 5A. We evaluated the posterior probability of theory of mind by marginalising over the bi-partition of fixed and theory of mind models, and it can be seen that the likelihood of the theory of mind model is substantially higher than the fixed model. Furthermore, the model with types *K*_1_ = 4 and *K*_2_ = 3 supervenes, yielding a 94.5% confidence that this is the correct model. The implicit densities used by the players on each others strategy *p*(*k*_2_|*y*,*k*_1_) and *p*(*k*_1_|*y*,*k*_2_) (see Equation 11) are exactly the same as in Figure 5A because the veridical model was selected.

Because we assumed the model is stationary over trials, the conditional confidence level increases with the number of trials; although this increase depends on the information afforded by the particular sequence. On the other hand, the posterior distribution over models tends to be flatter as the model-space expands because the difference between successive value-functions, 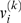 and 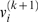 becomes smaller with increasing order. For the stag-hunt game in Figure 2, value-functions with *k*≥4 are nearly identical. This means that we could only infer with confidence that, *K_i_*≥5 (see Figure S1).

## Results

In this section, we apply the modelling and inference procedures of the preceding section to behavioural data obtained while real subjects played a stag-hunt game with a computer. In this experiment, subjects navigated a grid maze to catch stags or rabbits. When successful, subjects accrued points that were converted into money at the end of the experiment. First, we inferred the model used by subjects, under the known policies of their computer opponents. This allowed us to establish whether they were using theory of mind or fixed models and, under theory of mind models, how sophisticated the subjects were. Using Equation 10 we then computed the subjects' conditional densities on the opponent's strategies, under their maximum *a posteriori* sophistication.

### Experimental Procedures

**Figure 6:**
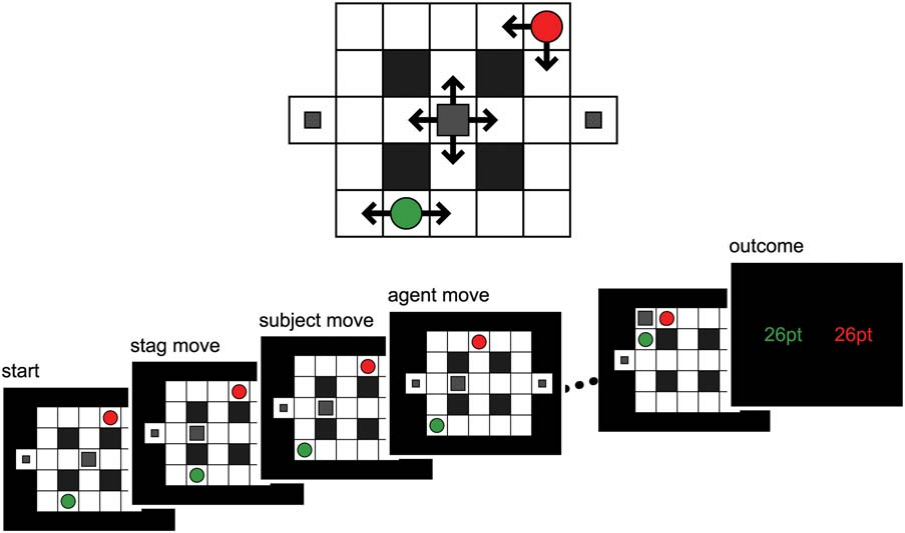
Stag-hunt game with two hunters: a human subject and a computer agent. The aim of the hunters (red and green circles) is to catch stag (big square) or rabbit (small squares). The hunters and the stag can move to adjacent states, while the rabbits are stationary. At each trial, both hunters and the stag move sequentially; the stag moved first, the subject moved next, and the computer moved last. Each round finishes when either of the hunters caught a prey or when a maximum number of moves had expired.

The subject's goal was to negotiate a two-dimensional grid maze in order to catch a stag or rabbit (Figure 6). There was one stag and two rabbits. The rabbits remained at the same grid location and consequently were easy to catch without help from the opponent. If one hunter moved to the same location as a rabbit, he/she caught the rabbit and received ten points. In contrast, the stag could move to escape the hunters. The stag could only be caught if both hunters moved to the locations adjacent to the stag (in a co-operative pincer movement), after which they both received twenty points. Note that as the stag could escape optimally, it was impossible for a hunter to catch the stag alone. The subjects played the game with one of two types of computer agents; A and B. Agent A adopted a lower-order (competitive) strategy and tried to catch a rabbit by itself, provided both hunters were not close to the stag. On the other hand, agent B used a higher-order (cooperative) strategy and chased the stag even if it was close to a rabbit. At each trial, both hunters and the stag moved one grid location sequentially; the stag moved first, the subject moved next, and the computer moved last. The subjects chose to move to one of four adjacent grid locations (up, down, left, or right) by pressing a button; after which they moved to the selected grid. Each move lasted two seconds and if the subjects did not press a key within this period, they remained at the same location until the next trial.

Subjects lost one point on each trial (even if they did not move). Therefore, to maximise the total number of points, it was worth trying to catch a prey as quickly as possible. The round finished when either of the hunters caught a prey *or* when a certain number of trials (15±5) had expired. To prevent subjects changing their behaviour, depending on the inferred number of moves remaining, the maximum number of moves was randomised for each round. In practice, this manipulation was probably unnecessary because the minimum number of moves required to catch a stag was at most nine (from any initial state). Furthermore, the number of ‘time out’ rounds was only four out of a total 240 rounds (1.7%). At the beginning of each round the subjects were given fifteen points, which decreased by one point per trial, continuing below zero beyond fifteen trials. For example, if the subject caught a rabbit on trial five, he/she got the ten points for catching the rabbit, plus the remaining time points: 10 = 15−5 points, giving 20 points in total, whereas the other player received only their remaining time points; i.e., 10 points. If the hunters caught a stag at trial eight, both received the remaining 7 = 15−8 time points plus 20 points for catching the stag, giving 27 points in total. The remaining time points for both hunters were displayed on each trial and the total number of points accrued was displayed at the end of each round.

We studied six (normal young) subjects (three males) and each played four blocks with both types of computer agent in alternation. Each block comprised ten rounds; so that they played forty rounds in total. The start positions of all agents; the hunters and the stag, were randomised on every round, under the constraint that the initial distances between each hunter and the stag were more than four grids points.

### Modelling Value Functions

We applied our theory of mind model to compute the optimal value-functions for the hunters and *stag*. As hunters should optimise their strategies based not only on the other hunter's behaviour but also the stag's, we modelled the hunt as a game with three agents; two hunters and a stag. Here state-space became the Cartesian product of the admissible states of all agents, and the payoff was defined on a joint space for each agent; i.e., on a |*S*_1_|×|*S*_2_|×|*S*_3_| array. The payoff for the stag was minus one when both hunters were at the same location as the stag and zero for the other states. For the hunters, the payoff of catching a stag was one and accessed only when both the hunters' states were next to the stag. The payoff for catching a rabbit was one half and did not depend on the other hunter's state. For the uncontrolled transition probabilities, we assumed that all agents would choose allowable actions (including no-move) with equal probability and allowed co-occupied locations; i.e., two or more agents could be in the same state. Allowable moves were constrained by obstacles in the maze (see Figure 6).

We will refer to the stag, subject, and computer as the 1st, 2nd, and 3rd agent, respectively. The transition probability at each trial is 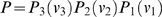. The *i*-th order value-function for the *j*-th agent, 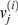, was evaluated through recursive solutions of the Bellman equations by generalising Equation 6 to three players
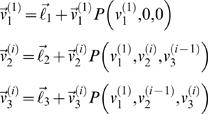
(13)Notice that the first agent's (stag's) value-function is fixed at first-order. This is because we assumed that the hunters believed, correctly, that the stag was not sophisticated. We used a convergence criterion of 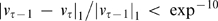 to calculate the optimal value-functions, using Equation 4. For simplicity, we assumed the sensitivity *λ* of each player was one. A maximum likelihood estimation of the subjects' sensitivities, using the observed choices from all subjects together, showed that the optimal value was *λ* = 1.6. Critically, the dependency of the likelihood on strategy did not change much with sensitivity, which means our inferences about strategy are fairly robust to deviations from *λ* = 1 (see Figure S2). When estimated individually for each subject, the range was 1.5≤*λ*≤1.8, suggesting our approximation was reasonable and enabled us to specify the policy for each value-function and solve Equation 13 recursively.

**Figure 7:**
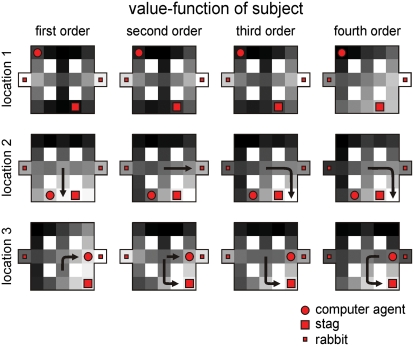
The optimal value-functions of the subjects for four different orders (columns) and for three different positions (rows). The circles are the computer agent's locations, and the big and small squares are the locations of the stags and the rabbits, respectively. Brighter colours indicate higher values.

The ensuing optimal value-functions of the subject, 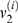, for *i* = 1,…,4 are shown in Figure 7. To depict the three-dimensional value-functions of one agent in two-dimensional state-space, we fixed the positions of the other two agents for each value-function. Here, we show the value-functions of the subject for three different positions of the computer and the stag (three examples of four value-functions of increasing order). The locations of the computer and stag are displayed as a red circle and square respectively. One can interpret these functions as encoding the average direction the subject would choose from any location. This direction is the one that increases value (lighter grey in the figures). It can be seen that the subject's policy (whether to chase a stag or a rabbit) depends on the order of value-functions *and* the positions of the other agents. The first-order policy regards the rabbits as valuable because it assumes that other agents move around the maze in an uncontrolled fashion, without any strategies, and are unlikely to help catch the stag. Conversely, if subjects account for the opponent's value-functions (i.e., using the second or higher order policies), they behave cooperatively (to catch a stag), provided the opponent is sufficiently close to the stag. Furthermore, with the highest order value-function, even if the other hunter is far away from the stag, the subject still tries to catch the stag (top right panel in Figure 7). For all orders of value-functions, the stag's value becomes higher than the rabbits', when the other hunter is sufficiently close to the stag (the middle row). However, interestingly, the policies here are clearly different; in the first-order function, value is higher for the states which are closer to the stag and the two states next to the stag have about the same value. Thus, if the subject was in the middle of the maze, he/she would move downward to minimize the distance to the stag. In contrast, in the second and higher-order functions, the states leading to the right of the stag are higher than the left, where the other hunter is. This is not because that the right side states are closer to another payoff, such as a rabbit. In fact, even when the other hunter is on the right side of the stag and very close to the rabbit, the states leading to the other (left) side are higher in the fourth-order function (bottom right panel). These results suggest that sophisticated subjects will anticipate the behaviour of other agents and use this theory of mind to compute effective ways to catch the stag, even if this involves circuitous or paradoxical behaviour.

### Modelling Strategy

**Figure 8:**
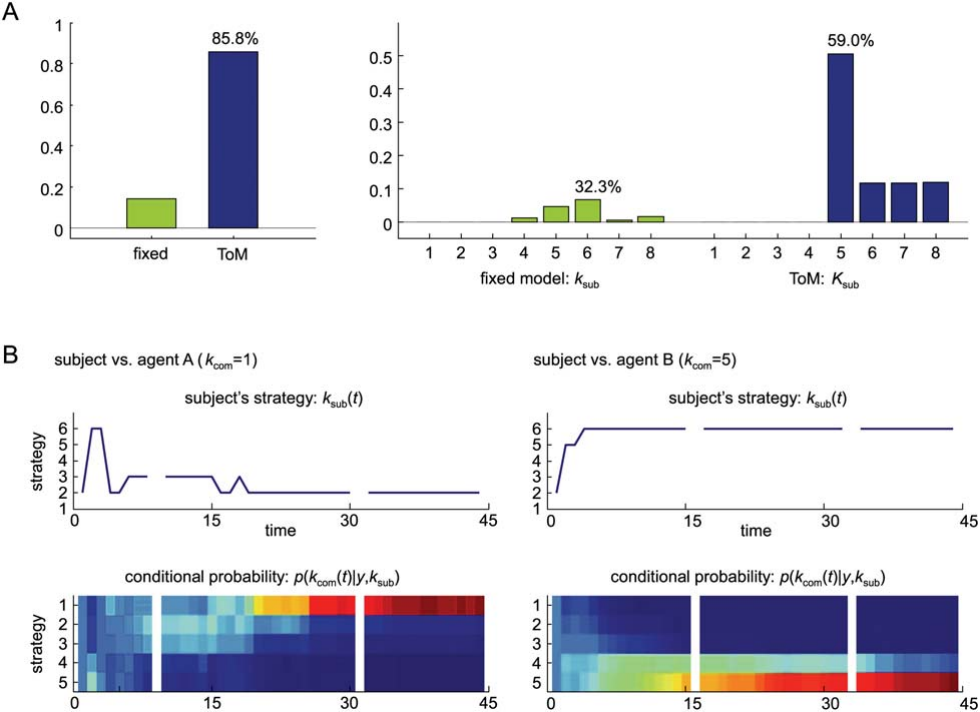
Results of the empirical stag-hunt game. (A) Model comparison. The posterior probabilities over the 16 models; eight fixed models with *k*_sub_ = 1,…,8 and eight theory of mind (ToM) models with *K*_sub_ = 1,…,8. The marginalized likelihood of the ToM models is higher than that of the fixed models (the left panel). Within the ToM model-space, the subject level is inferred as *K*_sub_ = 5. (B) The upper panels shows the inference on the subject's strategy over time in the sessions when the subjects played with the agent A (the left panel) and B (the right panel). The lower panels show the subject's densities on the computer's strategy.

Using these optimal value-functions, we applied the model comparison procedures above to infer the types of the subjects. We calculated the evidence for each subject acting under a fixed or theory of mind model using *k*_2_ = *k*_sub_ = 1,…,8 and *K*_2_ = *K*_sub_ = 1,…,8 and data pooled from all their sessions. We used the true order of the other players' policies for the model comparison; i.e., *k*_1_ = *k*_stag_ = 1 for the stag, *k*_3_ = *k*_com_ = 1 for the agent A and *k*_com_ = 5 for the agent B (Figure S3). Although, as mentioned above, these values do not affect inference on the subject's model. This entailed optimising *k*_sub_ and *K*_sub_ with respect to the evidence, for fixed models
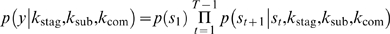
(14a)and theory of mind models
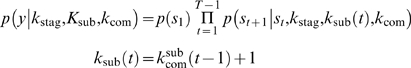
(14b)
Figure 8A shows the normalized posterior probabilities over the sixteen models. It can be immediately seen that the theory of mind model has a higher likelihood than the fixed model. Under theory of mind models, we inferred the most likely sophistication level of the subjects was *K*_sub_ = 5. This is reasonable, because the subjects did not have to use policies higher than *k*_sub_ = 6, given the computer agent policies never exceeded five. Among the fixed models, even though the likelihood was significantly lower, the optimal model, *k*_sub_ = 6, was inferred.

Using the inferred sophistication of the subjects, *K*_sub_ = 5, we then examined the implicit conditional density on their opponent's policy using Equation 11. Figure 8B show a typical example from one subject. The upper panels show the actual policies used when playing agent A (the left panel) and agent B (the right panel) and the lower panels show the subject's densities on the opponent's strategies. For both computer agents, the subject has properly inferred the strategy of the agent and plays at a level above it; i.e., the subject behaved rationally. This is a pleasing result, in that we can quantify our confidence that subjects employ theory of mind to optimise their choices and, furthermore, we can be very confident that they do so with a high level of sophistication. In what follows, we relate our game theory of mind to related treatments in behavioural economics and consider the mechanisms that may underpin sophisticated behaviour.

## Discussion

### Models in Behavioural Economics

Games with iterated or repeated play can differ greatly from one-shot games, in the sense that they engender a range of equilibria and can induce the notion of ‘reputation’, when there is uncertainty about opponents [17]. These games address important issues concerning how people learn to play optimally given recurrent encounters with their opponents. It has been shown that reputation formation can be formulated as a Bayesian updating of types to explain choices in repeated games with simultaneous moves [18],[19] and non-simultaneous moves [20]. An alternative approach to reputation formation is teaching [21]. In repeated games, sophisticated players often have an incentive to ‘teach’ their opponents by choosing strategies with poor short-run payoffs that will change what the opponents do; in a way that benefits the sophisticated player in the long run. Indeed, Camerer et al [22] showed that strategic teaching in their EWA model could select one of many repeated-game equilibria and give rise to reputation formation without updating of types. The crucial difference between these approaches is that in the type-based model, reputation is the attribute of a particular *player*, while in the teaching model, a *strategy* attains a reputation. In our approach, types are described in terms of bounds on strategy; the sophistication level. This contrasts with treatments that define types in terms of unobserved payoff functions, which model strategic differences using an attribute of the agent; e.g., normal or honest type.

Recursive or hierarchical approaches to multi-player games have been adopted in behavioural economics [23],[24] and artificial intelligence [25], in which individual decision policies systematically exploit embedded levels of inference. For instance, some studies have assumed that subject's decisions follow one of a small set of *a priori* plausible types, which include non-strategic and strategic forms. Under these assumptions, inference based on decisions in one-shot (non-iterated) games suggests that while policies may be heterogeneous, the level of sophistication may be equivalent to an approximate value of *k*; two or three. Camerer and colleagues [26] have suggested a ‘cognitive hierarchy’ model, in which subjects generate a form of cognitive hierarchy over each other's level of reciprocal thinking. In this model ‘*k*’ corresponds to the depth of tree-search, and when estimated over a collection of games such as the *p*-beauty game, yields values of around one and a half to two. Note that ‘steps of strategic thinking’ are not the same as the levels of sophistication in this paper. The sophistication addressed here pertains to the recursive representation of an opponent's goals, and can be applied to any iterated extensive form game. Despite this, studies in behavioural economics suggest lower levels of sophistication than ours. One reason for this may be that most games employed in previous studies have been one-shot games, which place less emphasis on planning for future interactions that rest on accurate models of an opponent's strategy.

In the current treatment, we are not suggesting that players actually compute their optimal strategy explicitly; or indeed are aware of any implicit inference on the opponent's policy. Our model is phenomenological and is designed to allow model comparison and predictions (under any particular model) of brain states that may encode the quantities necessary to optimize behaviour. It may be that the mechanisms of this optimization are at a very low level (e.g., at the level of synaptic plasticity) and have been shaped by evolutionary pressure. In other words, we do not suppose that subjects engage in explicit cognitive operations but are sufficiently tuned to interactions with con-specifics that their choice behaviour is sophisticated. We now pursue this perspective from the point of view of evolutionary optimization of the policies themselves.

### Prosocial Utility

Here, we revisit the emergence of cooperative equilibria and ask whether sophisticated strategies are really necessary. Hitherto, we have assumed that the utility functions ℓ*_i_* are fixed for any game. This is fine in an experimental setting but in an evolutionary setting, ℓ*_i_* may be optimised themselves. In this case, there is a fundamental equivalence between different types of agents, in terms of their choices. This is because exactly the same equilibrium behaviour can result from interaction between sophisticated agents with empathy (i.e., theory of mind) and unsophisticated agents with altruistic utility-functions. In what follows, we show why this is the case:

The recursive solutions for high-order value-functions in Equation 6 can be regarded as a Robbins-Monro scheme for optimising the joint value-functions over *N* players. One could regard this as optimising the behaviour of the group of players collectively, as opposed to optimising the behaviour of any single player. Once the joint value-functions have been optimized, such that 
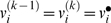
, they satisfy the Bellman equations
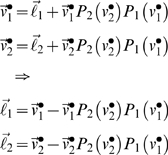
(15)However, these value-functions also satisfy
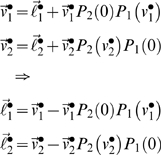
(16)This rearrangement is quite fundamental because we can interpret 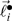 as optimal utility-functions, under the assumption that neither player represents the goals of the other. In other words, if two unsophisticated players were endowed with optimal utility-functions, one would observe exactly the same value-functions and behaviour exhibited by two very sophisticated players at equilibrium. These optimal 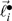 are trivial to compute, given the optimal value-functions from Equation 6; although this inverse reinforcement learning is not trivial in all situations (e.g., [27]). It is immediately obvious that the optimal utility 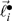 from Equation 16 has a much richer structure than the payoff ℓ*_i_* (Figure S4). Critically, states that afford payoff to the opponent now become attractive, as if ‘what is good for you is good for me’. This ‘altruism’ [28] arises because 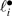 has become context-sensitive, and depends on the other player's payoff. An interesting example is when the optimised utility of state with a local payoff is greater when the opponent occupies states close to their payoff (see Figure S4). In other words, a payoff that does not depend on the opponent has less utility, when the opponent's payoff is low (c.f., guilt).

### Altruism and Inequity Aversion

This sort of phenomenon has been associated with ‘inequity aversion’. Inequity aversion is the preference for fairness [29] or resistance to inequitable outcomes; and has been formulated in terms of context-sensitive utility functions. For example, Fehr and Schmidt [5] postulate that people make decisions, which minimize inequity and consider *N* individuals who receive payoffs ℓ*_i_*. They then model the utility to the *j*-th player as
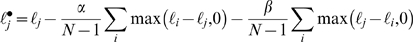
(17)where *α* parameterises distaste for disadvantageous inequality and *β* parameterises the distaste for advantageous inequality. Although a compelling heuristic, this utility function is an *ad hoc* nonlinear mixture of payoffs and has been critiqued for its rhetorical nature [30]. An optimal nonlinear mixture is given by substituting Equation 15 into Equation 16 to give
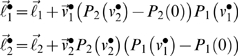
(18)These equalities express the optimal utility functions in terms of payoff and a ‘prosocial’ utility (the second terms), which allow unsophisticated agents to optimise their social exchanges. The prosocial utility of any state is simply the difference in value expected after the next move with a sophisticated, relative to an unsophisticated, opponent. Equation 15 might provide a principled and quantitative account of inequity aversion, which holds under rationality assumptions.

One might ask, what is the relevance of an optimised utility function for game theory? The answer lies in the hierarchal co-evolution of agents (e.g., [15],[31]), where the prosocial part of 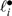 may be subject to selective pressure. In this context, the unit of selection is not the player but the group of payers involved in a game (e.g., a mother and offspring). In this context, optimising 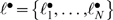 over a group of unsophisticated players can achieve exactly the same result (in terms of equilibrium behaviour) as evolving highly sophisticated agents with theory of mind (c.f., [32]). For example, in ethological terms, it is more likely that the nurturing behaviour of birds is accounted for by selective pressure on ℓ^•^ than invoking birds with theory of mind. This speaks to ‘survival of the nicest’ and related notions of prosocial behaviour (e.g., [33],[34]). Selective pressure on prosocial utility simply means, for example, that the innate reward associated with consummatory behaviour is supplemented with rewards associated with nursing behaviour. We have exploited the interaction between innate and acquired value previously in an attempt to model the neurobiology of reinforcement learning [35].

In summary, exactly the same equilibrium behaviour can emerge from sophisticated players with theory of mind, who act entirely out of self-interest and from unsophisticated players who have prosocial altruism, furnished by hierarchical optimisation of their joint-utility function. It is possible that prosocial utility might produce apparently irrational behaviour, in an experimental setting, if it is ignored: Gintis [33] reviews the evidence for empirically identifiable forms of prosocial behaviour in humans, (strong reciprocity), that may in part explain human sociality. “A strong reciprocator is predisposed to cooperate with others and punish non co-operators, even when this behaviour cannot be justified in terms of extended kinship or reciprocal altruism”. In line with this perspective, provisional fMRI evidence suggests that altruism may not be a cognitive faculty that engages theory of mind but is hard-wired and inherently pleasurable, activating subgenual cortex and septal regions; structures intimately related to social attachment and bonding in other species [36]. In short, bounds on the sophistication of agents can be circumvented by endowing utility with prosocial components, in the context of hierarchical optimisation.

Critically, the equivalence between prosocial and sophisticated behaviour is only at equilibrium. This means that prosocially altruistic agents will adapt the same strategy throughout an iterated game; however, sophisticated agents will optimise their strategy on the basis of the opponent's behaviour, until equilibrium is attained. These strategic changes make it possible to differentiate between the two sorts of agents empirically, using observed responses. To disambiguate between theory of mind dependent optimisation and prosocial utility it is sufficient to establish that players infer on each other. This is why we included fixed models without such inference in our model comparisons of the preceding sections. In the context of the stag-hunt game examined here, we can be fairly confident that subjects employed inference and theory of mind.

Finally, it should be noted that, although a duality in prosocial and sophisticated equilibria may exist for games with strong cooperative equilibria, there may be other games in which this is less clearly the case; where sophisticated agents and unsophisticated altruistic agents diverge in their behaviour. For example, in some competitive games (such as Cournot duopolys and Stackelberg games), a (selfish) understanding the other players response to payoff (empathy) produces a very different policy than one in which that payoff is inherently (altruistically) valued.

### Conclusion

This paper has introduced a model of ‘theory of mind’ (ToM) based on optimum control and game theory to provide a ‘game theory of mind’. We have considered the representations of goals in terms of value-functions that are prescribed by utility or rewards. We have shown it is possible to deduce whether players make inferences about each other and quantify their sophistication using choices in sequential games. This rests on comparing generative models of choices with and without inference. Model comparison was demonstrated using simulated and real data from a ‘stag-hunt’. Finally, we noted that exactly the same sophisticated equilibrium behaviour can be achieved by optimising the utility-function itself, producing unsophisticated but altruistic agents. This may be relevant ethologically in hierarchal game theory and co-evolution.

In this paper, we focus on the essentials of the model and its inversion using behavioural data, such as subject choices in a stag-hunt. Future work will try to establish the predictive validity of the model by showing a subject's type or sophistication is fairly stable across different games. Furthermore, the same model will be used to generate predictions about neuronal responses, as measured with brain imaging, so that we can characterise the functional anatomy of these implicit processes. In the present model, although players infer the opponent's level of sophistication, they assume the opponents are rational and that their strategies are pure and fixed. However, the opponent's strategy could be inferred under the assumption the opponent was employing ToM to optimise their strategy. It would be possible to relax the assumption that the opponent uses a fixed and pure strategy and test the ensuing model against the current model. However, this relaxation entails a considerable computational expense (which the brain may not be in a position to pay). This is because modeling the opponent's inference induces an infinite recursion; that we resolved by specifying the bounds on rationality. Having said this, to model things like deception, it will be necessary to model hierarchical representations of not just the goals of another (as in this paper) but the optimization schemes used to attain those goals by assuming agent's represent the opponent's optimization of a changing and possibly mixed strategy. This would entail specifying different bounds to finesse the ensuing infinite recursion. Finally, although QRE have become the dominant approach to modelling human behaviour in, e.g., auctions, it remains to be established that convergence is always guaranteed (c.f., the negative results on convergence of fictitious play to Nash equilibria).

Recent interest in the computational basis of ToM has motivated neuroimaging experiments that test the hypothesis that putative subcomponents of mentalizing might correlate with cortical brain activity, particularly in regions implicated in ToM by psychological studies [37],[38]. In particular, Hampton and colleagues [39] report compelling data that suggest decision values and update signals are indeed in represented in putative ToM regions. These parameters were derived from a model based on ‘fictitious play’, which is a simple, non-hierarchical learning model of two-player inference. This model provided a better account of choice behaviour, relative to error-based reinforcement learning alone; providing support for the notion that apparent ToM behaviour arises from more than prosocial preferences alone. Clearly, neuroimaging offers a useful method for future exploration of whether key subcomponents of formal ToM models predict brain activity in ToM regions and may allow one to adjudicate between competing accounts.

## Supporting Information

Figure S1 A. Log [Euclidean] distance between the value-functions in Figure 2B. B. Inference of opponent's types using the same simulated data used in Figure 5. Two players with asymmetric types *K*_1_ = 4 and *K*_2_ = 3. The left graph shows the likelihood over fixed models using *k*_1_,*k*_2_ = 1,…,6 and the right graph shows the likelihood of theory of mind models with *K*_1_,*K*_2_ = 0,…,5. The veridical model (dark blue bar) showed the maximum likelihood among 72 models.
(0.63 MB TIF)

Figure S2 Maximum likelihood estimation over the subject's type and payoff sensitivity. We used the models using *K*_sub_ = 0,…,5 and λ = 0.5,…,3.0 and data pooled from all subjects.
(0.72 MB TIF)

Figure S3 Inference of computer agent's policy: canonical inference using all subjects' data (A) and mean and standard deviation over six subjects (B). The order of agent A's policy is inferred as *k*_com_ = 1 and the agent B's order is inferred as *k*_com_ = 5.
(0.75 MB TIF)

Figure S4 The left panels show payoff functions for sophisticated agents who have theory of mind. The right panels show optimal utility functions for unsophisticated agents who do not represent opponent's goal: they assume opponent's policy is naïve.
(4.17 MB TIF)
