# Supplementary figures and images for: Game Theory of Mind
Source: PLoS Comput Biol. 2008 Dec 26;4(12):e1000254. doi: 10.1371/journal.pcbi.1000254 (PMC2596313; doi:10.1371/journal.pcbi.1000254)

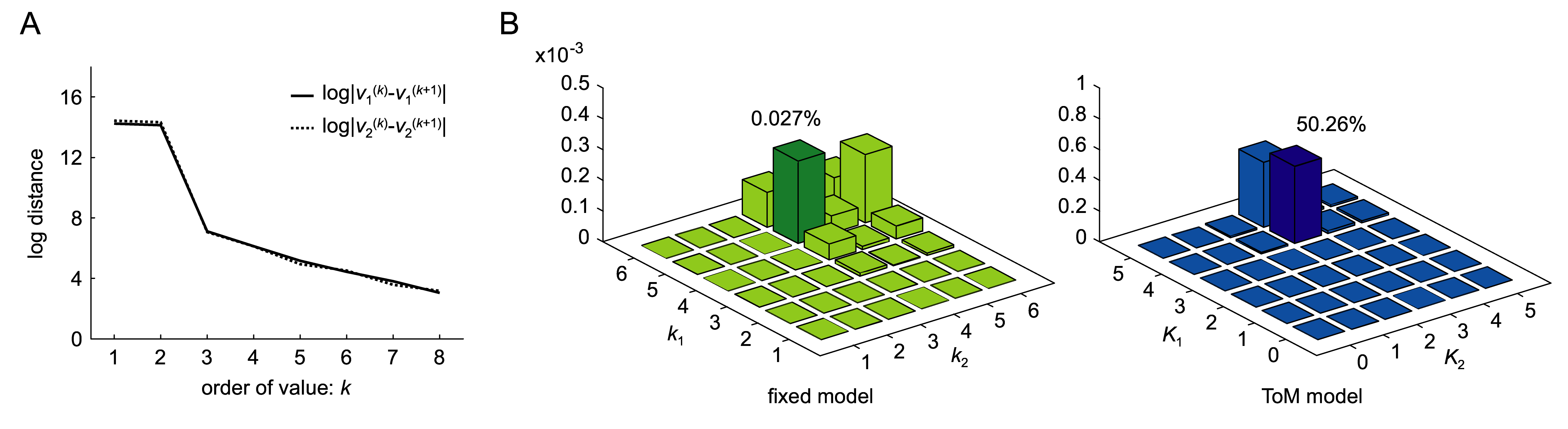

Supplement: Figure S1 — A. Log [Euclidean] distance between the value-functions in Figure 2B. B. Inference of opponent's types using the same simulated data used in Figure 5. Two players with asymmetric types K 1 = 4 and K 2 = 3. The left graph shows the likelihood over fixed models using k 1,k 2 = 1,…,6 and the right graph shows the likelihood of theory of mind models with K 1,K 2 = 0,…,5. The veridical model (dark blue bar) showed the maximum likelihood among 72 models. (0.63 MB TIF) [file pcbi.1000254.s001.tif]

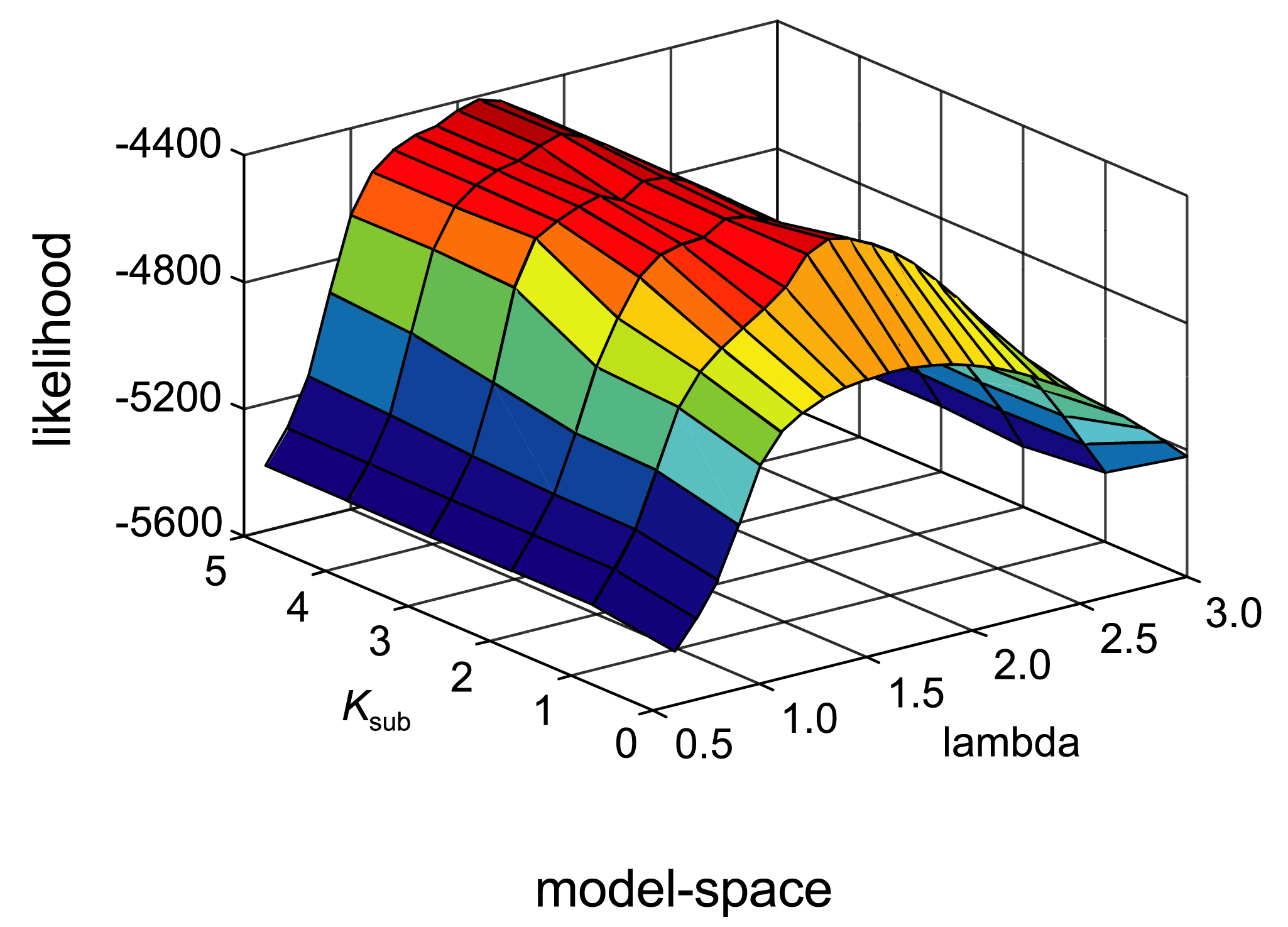

Supplement: Figure S2 — Maximum likelihood estimation over the subject's type and payoff sensitivity. We used the models using K sub = 0,…,5 and λ = 0.5,…,3.0 and data pooled from all subjects. (0.72 MB TIF) [file pcbi.1000254.s002.tif]

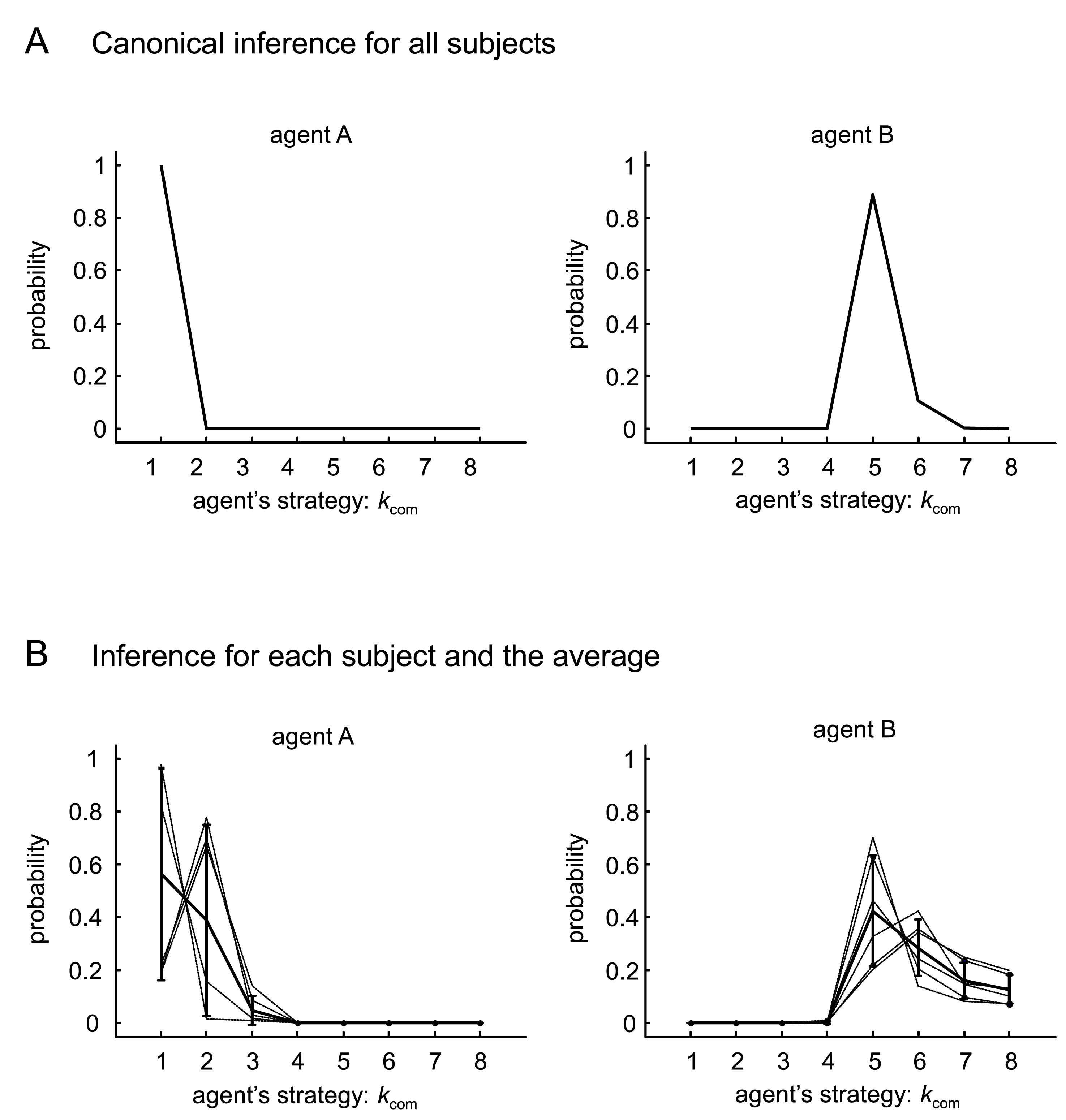

Supplement: Figure S3 — Inference of computer agent's policy: canonical inference using all subjects' data (A) and mean and standard deviation over six subjects (B). The order of agent A's policy is inferred as k com = 1 and the agent B's order is inferred as k com = 5. (0.75 MB TIF) [file pcbi.1000254.s003.tif]

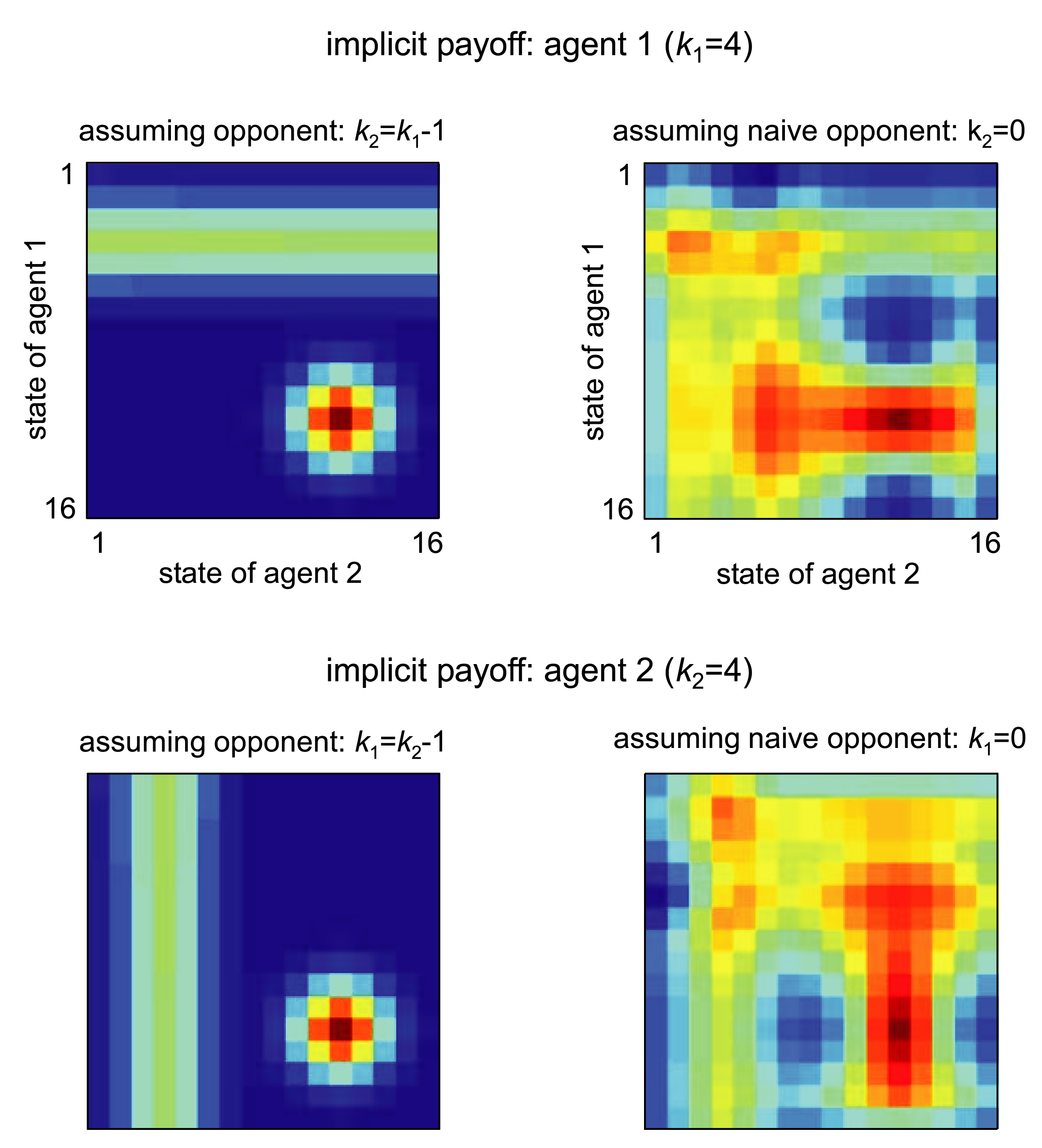

Supplement: Figure S4 — The left panels show payoff functions for sophisticated agents who have theory of mind. The right panels show optimal utility functions for unsophisticated agents who do not represent opponent's goal: they assume opponent's policy is naïve. (4.17 MB TIF) [file pcbi.1000254.s004.tif]
